# Isolation of bacteria-containing phagosomes by magnetic selection

**DOI:** 10.1186/1471-2121-9-35

**Published:** 2008-06-27

**Authors:** Per Lönnbro, Pontus Nordenfelt, Hans Tapper

**Affiliations:** 1 Division of Infection Medicine, Department of Clinical Sciences, Lund University, SE-221 84 Lund, Sweden

## Abstract

**Background:**

There is a growing awareness of the importance of intracellular events in determining the outcome of infectious disease. To improve the understanding of such events, like phagosome maturation, we set out to develop a versatile technique for phagosome isolation that is rapid and widely applicable to different pathogens.

**Results:**

We developed two different protocols to isolate phagosomes containing dead or live bacteria modified with small magnetic particles, in conjunction with a synchronized phagocytosis protocol and nitrogen cavitation. For dead bacteria, we performed analysis of the phagosome samples by microscopy and immunoblot, and demonstrated the appearance of maturation markers on isolated phagosomes.

**Conclusion:**

We have presented detailed protocols for phagosome isolation, which can be adapted for use with different cell types and prey. The versatility and simplicity of the approach allow better control of phagosome isolation, the parameters of which are critical in studies of host-bacteria interaction and phagosome maturation.

## Background

Phagocytosis and killing of microorganisms by phagocytes form an essential part of our innate immune system. The contact between the phagocyte and its prey triggers signaling to multiple intracellular events including cytoskeletal rearrangement, membrane traffic, and cytokine and chemokine responses (for review see [1]). Phagocytosis is important not only for killing of microorganisms, but also as a link between innate and acquired immunity by enhancing antigen presentation by dendritic cells [2,3].

Most knowledge regarding the maturation of a nascent phagosome into an antimicrobial phagosome comes from the study of macrophages. In neutrophils the process differs sufficiently as to still leave many questions unanswered [4]. In the neutrophil, granule-phagosome fusion is an integral part of phagosome maturation and a requirement for killing of ingested microorganisms. Accordingly, some intracellular pathogens have evolved means to disturb the normal maturation of the phagosome [5]. For instance, *Streptococcus pyogenes *bacteria of the M1 serotype can survive phagocytosis by neutrophils [6], and have been shown to interfere with the fusion of azurophilic granules with the phagosome [7].

Techniques for the isolation and analysis of phagosomes are important experimental tools in phagocytosis research. Current methods are dependent mainly on density-based ultracentrifugation as introduced by Wetzel and Korn in 1969 [8]. Such separation principles have been applied to latex bead-containing phagosomes from macrophages [9], and *Dictyostelium *[10]. Lührmann et al. used a similar technique to isolate bacteria-containing phagosomes [11]. However, using centrifugation, isolating phagosomes containing real bacteria is a lengthy and cumbersome process [12]. Introducing novel approaches, Russell et al. used iron-containing latex beads [13], and also performed magnetic isolation of mycobacteria-containing phagosomes using pre-loaded iron-dextran [14].

In this paper we present a method where the attachment of magnetic particles to the prey allows rapid and gentle isolation of bacteria-containing phagosomes.

## Results

### Overview of method

**Figure 1:**
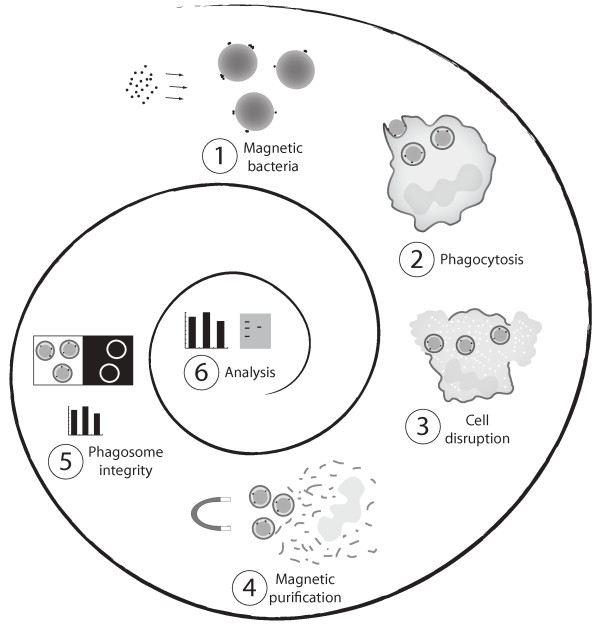
**Overview of method**. **1**. "Magnetic bacteria" are prepared by covalently attaching very small magnetite particles to the surface of the bacteria. This can be done in large batches. If dead bacteria are used the finished product may be stored for several weeks at 4°C. **2**. Synchronized phagocytosis of the magnetic bacteria is achieved through a 30-s centrifugation of a mixture of phagocytic cells and magnetic bacteria. This step may be repeated after resuspension to increase the interaction efficiency. Simultaneous phagocytosis of multiple samples can be performed using multi-channel pipettes in conjunction with either test tubes or microtiter plates. **3**. After completed presentation, free bacteria are washed away. Following an optional chase period, the suspension is then put on ice and pooled, and the buffer changed to an isotonic sucrose solution containing protease inhibitors and DNAse. The resulting suspension is put in a bomb cylinder and subjected to nitrogen cavitation (300 psi, 10 min) to disrupt the phagocytic cells. **4**. Aliquots of cell lysate are put into microtiter wells. Phagosomes are retrieved magnetically using a magnetic rod. Each well is probed several times to increase yield. **5**. Phagosome integrity is determined using direct fluorescent staining of a phagosome membrane marker and antibodies recognizing free or partially free bacteria. **6**. Isolated phagosomes are analyzed using immunofluorescence microscopy, flow cytometry, or immunoblot. Steps 2–5 can be achieved in less than 1 h.

The methods presented in this paper introduce refinements and novel approaches to several existing and proven techniques. The goal was an easy, rapid, gentle and generally applicable method for studying phagosome maturation in neutrophils. Our approach is summarized in Figure 1. The first step was to covalently attach very small magnetite particles to the surface of the bacteria. For this, we developed two different protocols; one primarily used with live bacteria and the other with dead. Bacteria made magnetic can be opsonized and bacterial aggregates can be removed by gentle centrifugation. Once the bacteria are ready for use, the phagocytes, in this case differentiated HL-60 cells, are harvested, washed and resuspended in cell medium. To achieve synchronized phagocytosis, the bacteria are then presented to the cells by a short centrifugation, which may be repeated to increase interaction efficiency (slightly compromising synchronization). After the presentation step, non-internalized bacteria are removed and a chase period at 37°C follows before the suspension is put on ice. In the cold, the buffer is changed to an isotonic sucrose buffer containing protease inhibitors and DNAse. This solution is put inside a bomb cylinder and subjected to nitrogen cavitation in order to disrupt the cells. Phagosomes are then retrieved magnetically. Phagosome integrity is determined by staining with both fluorescent annexin V and an anti-prey antibody (e.g. Cy3-labeled anti-human Fab fragments that label opsonizing human IgG), as positive and negative phagosome markers, respectively. Finally, phagosomes are analyzed by standard methods such as immunofluorescence microscopy, flow cytometry, or immunoblot.

### Preparing magnetic bacteria

**Figure 2:**
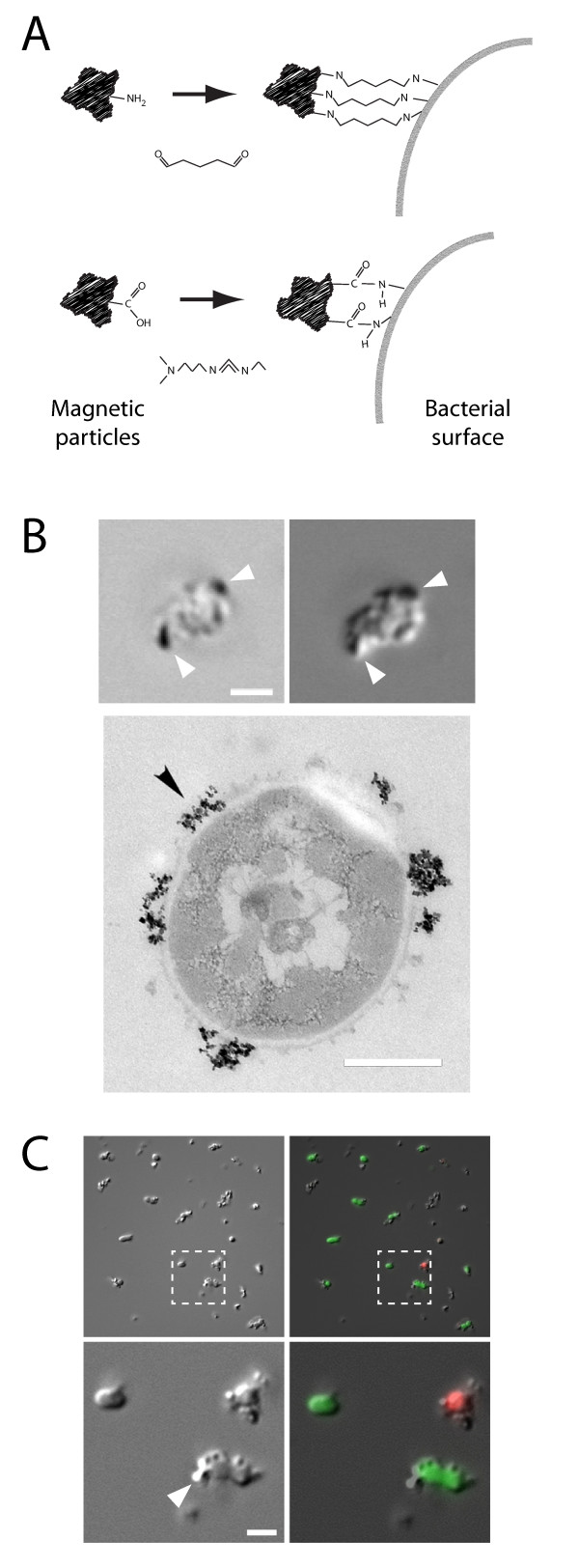
**Attachment of superparamagnetic particles to the surface of bacteria**. Panel A shows the two principles for covalent linkage of magnetic particles to bacteria. Top: glutaraldehyde can be used to create a pentyl bridge between amino group-exposing particles and the bacteria. Bottom: carbodiimide activation of carboxy group-exposing particles can be used to create peptide bonds with the bacteria. Panel B demonstrates the visualization of magnetite particles (arrowheads). Upper images: phase contrast and differential interference contrast, respectively; scale bar 1 μm. Lower image: electron micrograph depicting a bacterium (*S. pyogenes*) with attached magnetite particles; scale bar 0.5 μm. Panel C illustrates that, using the carbodiimide protocol, the attachment of particles (arrowhead) does not compromise the viability of bacteria, as determined by a BacLight Live-Dead kit (green = live, red = dead); scale bar 1 μm.

Central to the method is the ability to make bacteria susceptible to a magnetic field. For studies of phagosomal maturation, it is essential that this process will not change the bacteria in a way that influences the host cell-bacteria interaction. Covalent linkage of nanometer-scale superparamagnetic particles at a proper ratio should satisfy these conditions. In the following, we refer to bacteria artificially made superparamagnetic as "magnetic bacteria". Figure 2B shows an electron micrograph of a magnetic bacterium. The magnetic particles form clusters at the bacterial surface. These are also visible using phase contrast and differential interference contrast light microscopy, see top in Figure 2B. To obtain particles of sufficiently small size we have taken advantage of the fact that commercial magnetite particle preparations may have a wide size distribution. By centrifugation it is possible to remove larger particles and aggregates. We developed two different approaches for the covalent attachment of magnetite particles to the surface of bacteria. Both are based on protocols from Bangs Laboratories and are appropriate for beads/particles that have either amino or carboxyl groups on their surface. It should be possible to adapt the method for use with other commercially available or custom-made particles. Initial development of the method was performed using the amino variant, where glutaraldehyde is used as a cross-linking agent to form pentyl bridges between the particles and the bacterial surface, see top in Figure 2A. We obtained indirect evidence that the coupling procedure does not obstruct protein interactions at the bacterial surface to any significant degree. Wild-type M1 streptococci have a tendency to form aggregates due to the presence of M and H surface proteins, and this characteristic was retained after the bacteria had been subjected to our particle attachment protocol. Likewise, the lesser aggregation ability of a mutant strain lacking these surface proteins remained unchanged after particle coupling. After coupling, most of the bound particles remained attached to the bacteria after vortexing, needle-assisted shearing, or water-bath sonication.

Conjugation of magnetite particles to live bacteria was also performed, in this case using magnetite particles that exposed carboxyl groups. With carbodiimide as a carboxyl activating agent, carboxyl groups will react with primary amine groups to form peptide bonds, see bottom in Figure 2A. Using a water soluble form of carbodiimide, conjugation of the carboxyl magnetite particles to the primary amine groups of live *S. pyogenes *bacteria was achieved within one hour, in ordinary PBS buffer. In control experiments using carboxyl magnetite particles that had not been activated with the carbodiimide a much lower degree of particle binding to the bacteria was observed. As indicated by a fluorescent viability probe, bacterial membrane integrity was unaffected by the conjugation procedure, see Figure 2C. However, compared with the glutaraldehyde protocol, the particles appeared to be somewhat less resistant to dislodging from the bacteria by water-bath sonication or needle-assisted shearing.

### Magnetic purification

**Figure 3:**
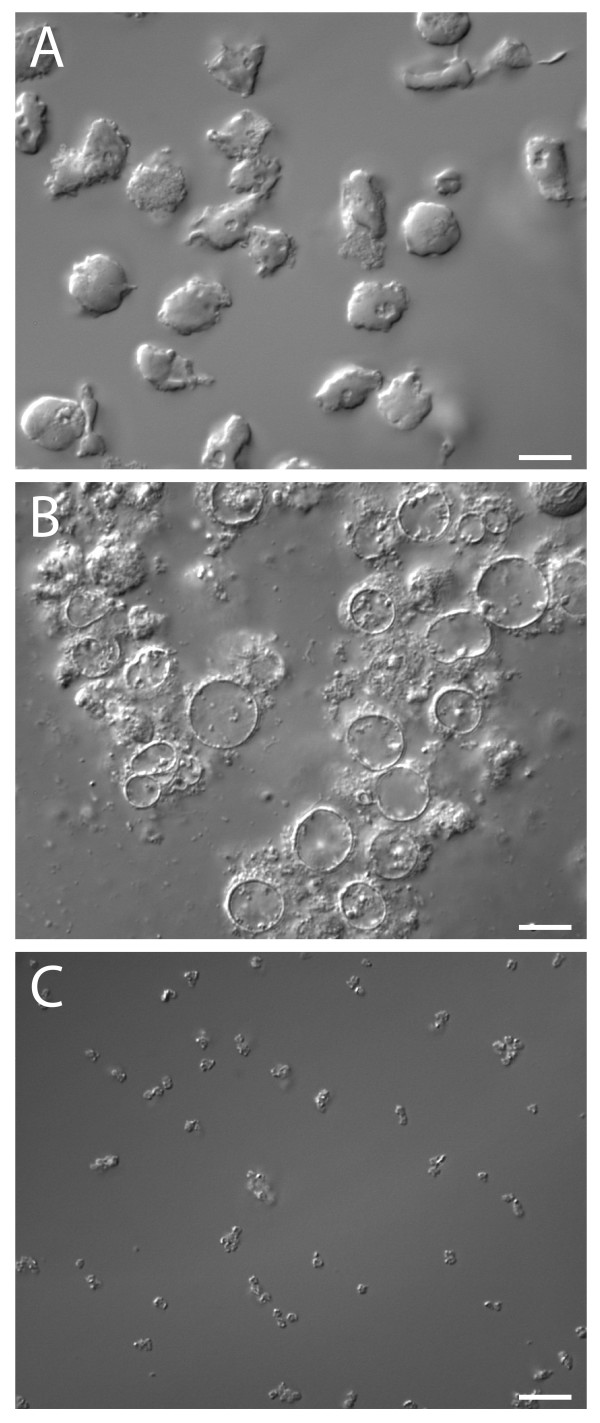
**Magnetic purification**. A shows intact HL-60 cells after phagocytosis of bacteria. B shows the same material after nitrogen cavitation, and C shows what can be retrieved from such a sample by one magnetic purification step (mostly phagosomes and free bacteria); scale bar 10 μm.

Having established efficient conjugation protocols, the magnetic bacteria were next used in a biological system. Neutrophils or differentiated HL-60 cells showed no difference in the interaction/uptake of modified bacteria in comparison with normal bacteria (data not shown). After phagocytosis, nitrogen cavitation was used to disrupt the cells. Figure 3 illustrates this and the effectiveness of magnetic separation. Figure 3A shows intact cells that have phagocytosed bacteria, and Figure 3B shows the sample appearance after nitrogen cavitation. Figure 3C shows the retrieved material after a single magnetic purification.

### Phagosome integrity

**Figure 4:**
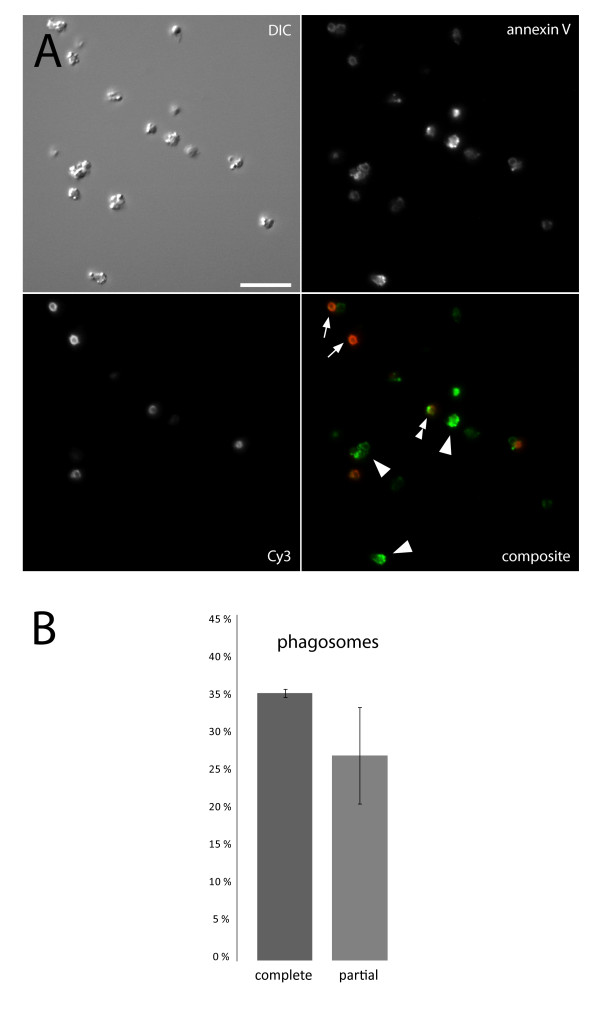
**Phagosome integrity**. In A, discrimination of free bacteria from intact and broken phagosomes is illustrated. Alexa 488-labeled annexin V (green) stains phagosomal membrane and Cy3-labeled anti-human Fab fragments (red) stains damaged phagosomes and free opsonized bacteria. Arrows indicate free bacteria, arrowheads intact phagosomes, and the double arrowhead shows a partial phagosome; scale bar 10 μm. B shows quantification of phagosomes from three separate experiments ± SEM.

To show the isolation of intact phagosomes, fluorescence microscopy was used. Annexin V binds to phosphatidyl serine, a lipid normally present in the inner leaflet of the plasma membrane and in the outer leaflet of the phagosomal membrane. Therefore, annexin V can be used as a marker for phagosomes. The impermeability of phospholipid membranes to most large molecules, such as antibodies, can also be utilized. To exemplify, an anti-human antibody can be used as a marker for non-sealed phagosomes and free bacteria when bacteria have been opsonized with human IgG. By taking a small sample of isolated phagosomes, adding calcium, Alexa 488-conjugated annexin V, and Cy3-labeled anti-human Fab fragments; the yield of intact phagosomes was determined, see Figure 4A. Any labeled anti-prey antibody can substitute for anti-human Fab fragments. This approach is quick and simple, and provides valuable information about the quality of the phagosome sample. The quantitation presented in Figure 4B shows that a substantial fraction of the isolated material consisted of intact or semi-intact phagosomes.

### Phagosome analysis

**Figure 5:**
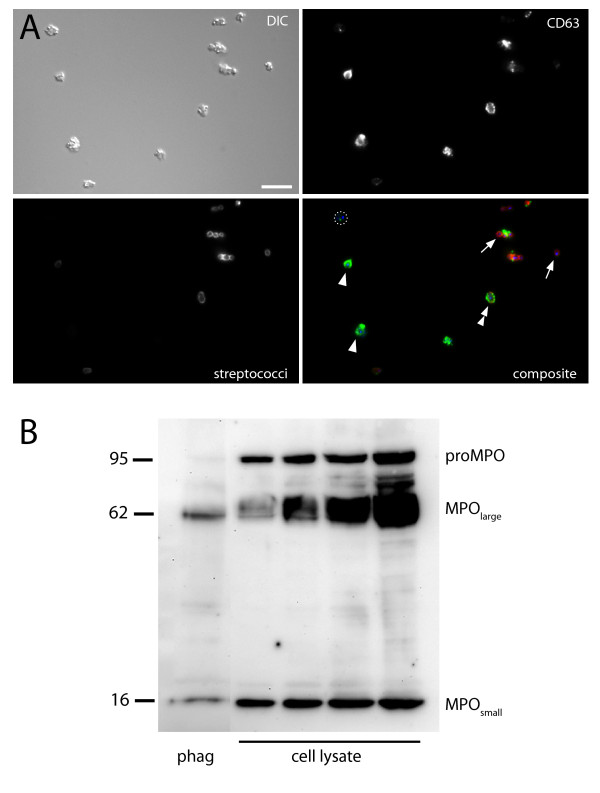
**Phagosome analysis**. Analysis of 15-min phagosomes, purified using the protocol for heat-killed bacteria. In A, immunofluorescence analysis of phagosomes is exemplified. Arrows show free bacteria, arrowheads intact phagosomes, double arrowheads broken phagosomes, and the dotted circle represents an intact phagosome negative for CD63 staining; scale bar 10 μm. Panel B shows Western blot analysis. Phagosomes (5·10^6^) and varying amounts of whole-cell lysates (4·10^4^, 1·10^5^, 2·10^5^, and 4·10^5^) were probed with an antibody against myeloperoxidase (MPO). Two mature-form fragments (MPO_large _and MPO_small_) and a precursor form (proMPO) can be detected.

Speed, ease of use, and the ability to use standard methods for analysis are the benefits of magnetic phagosome isolation. A particular advantage of the magnetic approach is that the isolated material can be concentrated in a gentle and rapid way. This is convenient for both immunofluorescence microscopy and Western blot. Figure 5A shows CD63 on isolated phagosomes. Free bacteria and semi-intact phagosomes can be identified by the use of an anti-prey antibody. Western blot analysis (Figure 5B) of phagosomes shows that only the active form of myeloperoxidase (MPO_large _and MPO_small_) is present in isolated phagosomes. Since this form of MPO is normally present in azurophilic granules, this shows that delivery of azurophilic granule content to the phagosomes has taken place. In contrast, one of the MPO proforms [15,16] is notably absent from the phagosomes. The fact that proMPO is normally present in the synthetic apparatus of the cells suggests that our isolated phagosomes are of high purity, in particular lacking ER/Golgi contamination.

## Discussion

Several important human pathogens can use various mechanisms to manipulate the maturation of phagosomes. Understanding the dynamic host-pathogen interactions occurring inside host cells is not a trivial challenge, and there is a need to develop novel technical solutions to be able to better study phagosome maturation.

The most commonly used technique for phagosome maturation studies is whole-cell fluorescence microscopy, but its results can be difficult to analyze and quantitate [17]. This is especially true for smaller-sized cells, such as neutrophils. Transfection with fluorescent proteins in neutrophils is not an easy experimental approach either, since these cells are terminally differentiated and have a short life span. Our magnetic method was not developed to analyze intact cells, but rather for the study of isolated phagosomes by microscopy, flow cytometry, or biochemical assays.

So far, the techniques most often used to purify phagosomes are based on differential ultracentrifugation of latex beads [8]. Using this inert "model prey" allows investigation of model phagosome maturation, and many important findings have been made using this approach. However, the long centrifugation time is a problem when investigating dynamic processes such as phagosome maturation, even if performed at 4°C. Speed and ease of use are obvious benefits of using magnetism as a selection principle, since separation steps are performed in terms of minutes rather than hours.

The use of magnetism as a tool in cell biology is not new, and has been successfully employed in cell separation [18] as well as in protein chemistry [19]. Using a different approach, magnetic isolation of bacteria-containing phagosomes has previously been performed by Pethe et al. [14]. Our approach differs in several aspects from their protocol. Most importantly, since our magnetic phagosome purification technique does not rely on sequential endocytosis, i.e. the iron preloading followed by phagocytosis of the actual prey, isolation of early or maturation-halted phagosomes is possible and does not require fusion of other endocytic compartments with the forming phagosome. Another benefit of the protocol is that, with the exception of the disruption step, it is readily adaptable for automation using commercially available robot systems. This is especially true for the magnetic separation steps where the standard microtiter format is already in use. The method, as described in the present publication, is a further development of a separation protocol that was published earlier as part of a book chapter [20]. Besides being optimized with respect to parameters such as cell disruption, phagocytosis, and centrifugation speeds, several other major improvements have been added. These include an entirely new protocol for the attachment of magnetic particles to live bacteria and the introduction of steps to minimize the influence of bacterial aggregation. Also, a quick and easy assay to monitor the integrity of each phagosome preparation has been added to the protocol. Taken together, these refinements have greatly increased the reproducibility of the method, and also increased the obtainable phagosome yield and purity.

The efficient removal of contaminants is difficult to achieve in most methods of phagosome isolation [21], and in this respect our method is probably no exception. Particularly for biochemical analysis, purity is critically important. In our experience, the most critical steps in magnetic phagosome isolation are efficient presentation and phagocytosis, removal of non-internalized bacteria, and efficient and controlled cell breakage. For magnetic separation, purity largely relies on the effectiveness of the cell disruption. Therefore, optimization of nitrogen cavitation parameters, such as pressure, equilibration time and cell density, is essential. Also, as pointed out by Lührmann et al., degradation of DNA by the addition of endonuclease improves the yield during purification of phagosomes [11]. How our protocol compares to other techniques in terms of purity remains to be demonstrated.

The data presented here is based on the use of streptococci as phagocytic prey, but it should be possible to apply the method to other types of prey as well, independently of size, shape or density. In our experiments, phagocytosis was performed by differentiated HL-60 cells, but human neutrophils or other phagocytic cells, purified according to standard methods [22], may also be used, without modification of the magnetic isolation protocol.

## Conclusion

In this paper we present a method whereby the use of magnetic particles makes it possible to magnetically isolate phagosomes containing any type of prey.

## Methods

### Bacteria

The *Streptococcus pyogenes *AP1 strain of the M1 serotype was obtained from the World Health Organization Streptococcal Reference Laboratory in Prague, Czech Republic. From this strain the mga regulon deficient mutant BMJ71 was generated by Kihlberg et al. [23] using transposon mutagenesis. This mutant lacks expression of M protein, protein H, protein SIC and C5a peptidase. A few colonies were seeded in 10 ml of autoclaved, freshly prepared Todd Hewitt broth (with 5 μg/ml tetracyclin added) and incubated overnight at 37°C and 5% CO_2_. An aliquot of the bacteria was inoculated into 10 ml and grown to logarithmic phase (as estimated by OD measurements at 620 nm), then washed three times in autoclaved PBS. For experiments using dead bacteria, these were heat-killed at 80°C for ten minutes in a heat block, followed by a rapid cooling-down of the samples in ice water.

### Magnetic bacteria

BioMag BM546 (Bangs Laboratories, Inc., Fishers, IN) is an aqueous suspension of irregularly shaped superparamagnetic particles, nominal mean diameter 1.5 μm, composed of >90% magnetite (Fe_3_O_4_) and an inert silane coating containing free amino groups. Microscopic inspection revealed a considerable variation in size. By centrifugation (1,000 *g*, 30 s, fixed angle) of 50 μl stock suspension diluted with water to 1,000 μl, we were able to collect 900 μl of supernatant containing a particle fraction with a maximal diameter of less than a fifth of the original mean value, as estimated by light microscopy. Using electron microscopy, we observed that the isolated particles are clusters of nanoscale magnets, found in aggregates of approximate size 50–100 nm.

Our coupling procedure is a modification of a commercial protocol for protein solutions (BioMag Data Sheet #546, Bangs Laboratories). The supernatant containing the isolated particles was put on a magnetic Eppendorf rack (Dynal MPC-M, Dynal A.S., Oslo, Norway), where the particles were allowed to migrate, perpendicular to gravity, to the magnetic wall for 10 minutes. The solution was then exchanged for 1 ml of coupling buffer (0.01 M pyridine, pH 6.0), by carefully aspirating from the bottom of the tube. This step was repeated three times, after which the particles were treated with a cross-linking agent in the form of 5% glutaraldehyde in coupling buffer, with an addition of 0.05% Tween-20. The mixture was put on a rotator (18 rpm) for a three-hour incubation at room temperature. This was followed by fourfold washing in coupling buffer.

The next step brought together the pretreated particles with an appropriate amount of heat-killed streptococci. The glutaraldehyde, bound during the pretreatment to the amino groups of the BioMag particles, could then react with amino groups on the bacteria, forming pentyl bridges between the particles and the bacteria. To achieve a particle coverage of the bacterial surface sufficiently sparse as to leave much of the cell wall unobstructed, see Figure 2B, an empirically tested ratio of the number of particles and bacteria was used. The heat-killed bacteria were centrifuged (12,000 *g*, 6 min) and resuspended in coupling buffer. Thereafter, the bacterial suspension was mixed with the pretreated BioMag subfraction in a sterile Eppendorf tube, followed by a 30 s sonication. The reaction mixture was then transferred to a cold-storage room and put on a rotator (11 rpm) for overnight incubation.

Next day, the suspension was separated for 10 minutes on the magnetic rack, after which the superparamagnetic bacteria were resuspended in quenching solution (1 M glycine, 1% heat-shock fractionated bovine serum albumin (BSA), pH 8.0), briefly sonicated, and then put on a rotator (18 rpm, room temperature) for 30 minutes. Finally, the superparamagnetic bacteria were briefly sonicated and then washed in 4 × 1 ml PBS (including 0.05% BSA). They were resuspended in 1 ml of buffer and stored at 4°C.

As an alternative, intended for conjugation to live bacteria, BioMag carboxyl magnetite particles (BM570, Bangs Laboratories) were used. The particles were prepared by centrifugation (1,000 *g*, 30 s, fixed angle) of the stock suspension diluted in water to 1,000 μl, and then collecting 900 μl of the supernatant. Using the magnetic rack, the magnetite particles were washed twice in 0.1 M MES buffer (2-(*N*-morpholino)ethanesulphonic acid), pH 5.2. Following addition of 4 mg EDAC (1-ethyl-3-(3-dimethylaminopropyl)carbodiimide), the suspension was incubated on a rotator (12 rpm) for 15 minutes at RT. The chemically activated particles were washed twice in PBS, pH 7.4, using magnetic separation. Particles were then mixed, at an empirically selected ratio, with 0.5·10^9 ^live bacteria in an Eppendorf tube at a total volume of 1 ml PBS, and incubated at 37°C on a rotator (12 rpm) for 30 min. This was followed by blocking in 1% BSA in PBS at RT for 30 min on a rotator. Larger aggregates were then removed by centrifugation (200 *g*, 1 min, swing-out). Finally, the viability of the particle-conjugated bacteria was checked using LIVE/DEAD BacLight Bacterial Viability Kit (Invitrogen, Copenhagen, Denmark). This method exploits the different membrane permeabilities of two fluorescent dyes, SYTO 9 and propidium iodide. SYTO 9 labels all bacteria green, whereas propidium iodide labels bacteria with a compromised membrane red.

### Cell differentiation

The human promyelocytic leukemia cell line HL-60 can be differentiated into a neutrophil-like phenotype by means of chemical agents [24]. The acquired characteristics include phagocytic and microbicidal ability, and the presence of Fc-receptors and azurophilic granules. In our model system we used all-trans retinoic acid (ATRA) as an inducer of neutrophil differentiation, in accordance with the protocol of Breitman et al. [25]. Briefly, HL-60 cells were seeded at 0.3·10^6^/ml in L-glutamine-containing RPMI 1640 medium (PAA Labs, Pasching, Austria), supplemented with 10% fetal bovine serum (Gibco) and 1 μM ATRA (Sigma), and incubated in 5% CO_2 _atmosphere at 37°C, followed by harvesting after four or five days. No antibiotics were used. The viability of the differentiated cells was typically in the range of 75–80%, as determined by Trypan blue exclusion.

### Phagocytosis

Prior to phagocytosis, neutrophil-differentiated HL-60 cells were gently centrifuged (145 *g*, 5 min, swing-out) at room temperature in Falcon 50-ml tubes and resuspended in Na medium (containing 5.6 mM glucose, 127 mM NaCl, 10.8 mM KCl, 2.4 mM KH_2_PO_4_, 1.6 mM MgSO_4_, 10 mM Hepes, and 1.8 mM CaCl_2_; pH adjusted to 7.3 with NaOH). The magnetic bacteria were opsonized with human IgG (Sigma, 1 mg/ml in Na medium) for 30 min at 37°C. Before presentation, the bacterial suspension was briefly sonicated and centrifuged (200 *g*, 2 min, swing-out) to remove aggregates. Cells and bacteria were mixed at a ratio of 1:5 by adding the cells to microtubes preloaded with opsonized bacteria, making up a final volume of about 250 μl per sample. The resulting suspensions were equilibrated at 37°C for 1 minute in a thermostatted water bath. For an efficient presentation, no more than 5·10^6 ^cells were used per test tube. To synchronize phagocytosis, the thermally equilibrated bacteria and cells were actively brought into contact with each other by means of a short centrifugation (12,000 *g*, 30 s, fixed angle; note that a gentler centrifugation can be performed, but at the cost of less efficient synchronization). Immediately after this, the pellets were resuspended and transferred back to the water bath for 30 s after which the process was repeated to increase interaction. The presentation step was halted by placing the samples on ice. After presentation, the samples were pooled, washed three times (200 *g*, 2 min, swing-out) to remove extracellular bacteria and finally resuspended in 1 ml Na medium for varying chase periods at 37°C.

### Nitrogen cavitation

The cells were centrifuged (200 *g*, 2 min, swing-out) and resuspended in 1 ml of cold isotonic protease-inhibitor buffer (0.25 M sucrose, 10 mM HEPES, 3 mM MgCl_2_·6H_2_O; Complete Mini EDTA-free, Roche Diagnostics GmbH, Mannheim, Germany; 1 tablet per 10 ml solution; Benzonase endonuclease, Merck KGaA, Darmstadt, Germany; 250 U per 20·10^6 ^cells). The nitrogen cavitation was carried out in a pressurized cell disruption bomb (4639, Parr Instrument Company, Moline, IL), the physical parameters chosen by Borregaard et al. [26] and Ballinger et al. [27] being used as guidelines. Following transfer of a 1-ml sample to the sample compartment of the bomb, the nitrogen partial pressure was increased to 300 psi, at which pressure the inert gas was allowed to dissolve in the cell suspension for ten minutes. A rapid release of the pressure then leads to the disruption of the cells. The eluate was led through a thin latex tube, attached to the elution valve, and was collected in a 50-ml Falcon tube equipped with a parafilm splash shield. In between samples, the bomb cavity and latex tube were thoroughly rinsed with deionized water.

### Magnetic purification of phagosomes

The cavitation samples were kept on ice for 5–15 min to allow the endonuclease to degrade any free DNA. The sample was then divided into 200-μl microtiter wells (Low-bind). To keep the temperature at 4°C, the plate was placed on an aluminum block in ice water. Magnetic separations of the samples were carried out using a PickPen magnetic rod and removable silicon tip covers (Bio-Nobile, Turku, Finland). To collect magnetic material, the tip is gently dipped into each well, kept submerged for 1–2 min, and then transferred to a new well containing wash solution. Keeping the silicon tip in solution, the magnetic rod is retracted, leading to the release of the material. The procedure is repeated 2–4 times.

### Phagosome integrity

The isolated phagosomes were analyzed by taking a 10-μl sample and adding CaCl_2 _(3.2 mM), Alexa Fluor 488-conjugated Annexin V (1:1,000, Invitrogen), Cy3-conjugated anti-human Fab fragments (1:1,000, Jackson Immunoresearch Laboratories, Inc., Suffolk, UK) and incubating for 5 min. At least 100 phagosomes/bacteria were characterized as free bacteria (red ring only), broken phagosomes (green and red ring) or intact phagosomes (green ring only).

### Immunofluorescence microscopy

Evaluation of the fusion of phagosomes with azurophilic granules was carried out by fluorescence microscopy using an antibody against CD63 and an antibody against streptococci. After allowing phagocytosis for 15 min, the phagosomes were isolated and incubated with blocking medium (Na medium with 1% BSA and 5% goat serum) for 15 min. The phagosomes were moved to a well containing anti-streptococcal antibody (1:1,000, goat) in blocking medium and incubated for 30 min on ice. This was followed by fixation with 1% PFA in Na medium for 15 min at 4°C, followed by 45 min incubation at room temperature. Next, phagosomes were washed twice in blocking buffer, and then permeabilized during a 30-min incubation at room temperature in permeabilization medium (blocking medium supplemented with 0.02% Triton X-100, 0.2% Tween-20, and 1 mg/ml human IgG). The primary mAb against CD63 (Santa Cruz BioTechnology, Santa Cruz, CA) was diluted in permeabilization medium at a ratio of 1:600 and 1:800, respectively. After an overnight incubation at 4°C, cells were washed twice in permeabilization medium before incubation (60 min at room temperature) with the Alexa Fluor 488 anti-mouse secondary antibody and the Alexa Fluor 594 anti-rabbit secondary antibody, both used at a final dilution of 1:1,200. Following two washes in Na medium, the samples were resuspended in 150 μl Na medium, adhered to poly L-lysine (MW 150,000, Sigma) coated glass cover slips for 30 min (assisted by magnets), and then mounted using ProLong Gold antifade reagent (Invitrogen). Visual inspection and recording of images were performed using a Nikon Eclipse TE300 inverted fluorescence microscope equipped with a Hamamatsu C4742-95 cooled CCD camera, using a Plan Apochromat 100× objective with numerical aperture of 1.4.

### Western blot

SDS-PAGE was a modification of the protocol of Laemmli [28] made according to instructions for NuPAGE gels (4–12% Bis-Tris, Invitrogen) and PVDF membranes (Millipore). The membranes were probed using antibodies against myeloperoxidase (1:5,000,[29]). Western blots were developed with Super Signal West Dura Extended (Pierce).

### Electron microscopy

Samples for electron microscopy were prepared by pelleting approximately 4·10^8 ^bacteria at 4°C immediately after addition of fixative (1.5% PFA and 1.5% glutaraldehyde in 0.15 M sodium cacodylate buffer, pH 7.4). After incubation at room temperature for 1 hour, the fixed pellets were postfixed for 2 h at 4°C in 1% osmium tetroxide in sodium cacodylate buffer, subsequently dehydrated in a series of ethanol steps, and then further processed with acetone for Epon embedding. Sections were cut with a microtome and mounted on Formvar coated copper grids. The sections were postfixed with uranyl acetate and lead citrate and examined under the electron microscope.

## Authors' contributions

PL and PN contributed equally to this work. HT initiated the project. PL conceived the idea of covalently attaching magnetite particles to streptococci, and set up a magnetic phagosome isolation protocol based on this. Further optimization of the method was mainly carried out by PN. All experimental work was performed by PL and PN. PN played a major role in drafting the text and figures. HT, PL and PN jointly finalized the manuscript. All authors have read and approved the final manuscript.
